# On Infantile Paralysis

**Published:** 1852-12

**Authors:** M. Rilliet


From the Archives Generales de Medecine.

*On Infantile Paralysis, by* M. RILLIET.

In an essay by M. Rilliet on "Essential Paralysis of Infants," we find a large amount of interesting matter, the most practical portion of which we shall endeavor to condense.

1. *Definition.*—The author applies the term "Essential Paralysis" to more or less complete loss of power, with or without loss of sensation, and unaccompanied by any signs of lesion of the nervous centres. This form of paralysis is often incurable, but does not of itself shorten life, for which reason it is difficult to find its structural causes; for even if the spinal marrow or brain shall exhibit certain lesions, it becomes doubtful whether they are not the results, rather than the causes, of the paralysis.

2. *Seat and Mode of Attack.*—Essential paralysis occurs in three different ways: sometimes it attacks the patient suddenly in its highest degree, and without obvious cause; at other times it is preceded by cerebral disturbance and convulsions, as during dentition, but in these cases, also, the loss of power is sudden and not progressive. Thirdly, it may approach gradually.

When paralysis is sudden, and not preceded by cerebral symptoms, it mostly appears in the upper extremities. An infant goes to bed well, and next morning it is found paralysed in one arm. Another sits on the damp ground, and one leg is suddenly discovered to have lost the power of motion. At the same time there is no perceptibled erangement of the general health.

When cerebral symptoms precede, these usually consist of somnolence, strabismus, dilatation of the pupil, and headache. These symptoms speedily subside. At other times violent and repeated convulsions occur; these cease, and paralysis is found to have supervened. Paralysis sometimes succeeds chorea, as in cases cited by Drs. Kennedy and Lee. It also appears in the course of exanthematic fevers, and is then generally discovered for the first time when convalescence commences.

3. *Symptoms and Progress.*—In whatever way infantile paralysis commences, it presents two periods,—one, that of paralysis, the other, that of atrophy. In some fortunate cases the disease does not proceed to the latter extent. The symptoms vary with the part affected. If the arm is the seat of the disease, it hangs lifeless, and if lifted falls again to the side. The paralysis is sometimes complete, at other times limited to certain sets of muscles. In some instances the fingers are flexed upon the thumb.

So also in the lower extremity the loss of power may be complete or partial. If the child does not walk, it kicks the sound leg about, while the other lies motionless. The paralysed limb is not the seat of any pain. The color and temperature of the skin are often normal. The sensation is generally intact. In fact, the paralysis of motion constitutes the entire malady.

The progress of the disease is not always the same; it may disappear completely and rapidly, or it may persist with or without amelioration. In the latter case, sooner or later, the second period or that of atrophy commences. This is marked by diminution of temperature, wasting of the muscles, and arrest of growth of all the structures together, so that the limb is perceptibly smaller and shorter than the other. In proportion as the temperature diminishes, so does the skin change color, becoming more and more livid.

Fresh observations on the conditions of the vessels of paralysed limbs are necessary, but it is obvious that these tubes are involved in the general atrophy. The pulse at the wrist is, in some cases, scarcely to be felt.

As a farther consequence of infantile paralysis, the spinal column becomes variously distorted, and the limbs themselves may be deformed. Thus, in paralysis of the arm, and atrophy of the deltoid, the head of the humerus may be completely dislocated, the weight of the limb stretching the capsular ligament, until it allows the head of the bone to glide out of the glenoid cavity. Such cases are described by West, and one is reported by the author of the present memoir.

Heine has described deformity of the lower limbs thus paralysed, consisting of flexion of the thigh upon the pelvis, and of the leg upon the thigh. The paralysis of one set of muscles, and antagonistic contraction of others, give rise to the different varieties of club-foot.

5. *Causes.*—Authors agree that this form of paralysis is more common in the first and second years of life than subsequently. In two-thirds of the cases on record the child was between the ages of six months and two years. However, West, Kennedy, and Heine have known the disease to attack children as late as five, six, and seven years of age. Sex has no influence on the disease. It is more likely to occur, in the opinion of some writers, in robust and well-formed, than in feeble and ill-nourished children; but such is not the author's experience. Among the occasional causes may be mentioned chills and blows.

6. *Diagnosis.*—The diagnosis of paralysis is not difficult, but it is not always easy to say whether the palsy is essential, or symptomatic of lesion of the nervous centres, unless the symptoms of the latter diseases have been well marked. The diseases of the brain which commence in convulsions at the age most liable to essential paralysis, are meningitis, tubercular hydrocephalus, and meningeal apoplexy. The two former diseases are generally accompanied by disturbance of the sensorium, which are not seen in simple paralysis, and are moreover generally mortal. The same may be said of meningeal apoplexy, which is moreover commonly followed by tonic contraction of the limb. M. Ozanam believes that meningeal apoplexy is the cause of all the cases of paralysis which are preceded by convulsions, but he does not adduce a single fact in support of his theory.

This form of paralysis can be confounded with diseases of the hip only by a very careless observer, and form the progressive muscular atrophy mentioned by M. Arau, it is distinguished by the latter being an affection peculiar to adult life.

7. *Treatment.*—West and Kennedy trust to purgatives and tonics, but allude entirely to the treatment of the stage of simple paralysis. Heine goes deeper into the subject, and endeavors to remedy the stage of atrophy also. The indications of treatment proposed by him are:—

1. To awaken the nervous power of the spinal marrow and nerves of the affected limb. 2. To restore the deformities by orthopedic measures. 3. To invigorate the constitution.

For the fulfilment of the first indication he gives nux vomica internally, and applies it in frictions along the spine. Electricity has failed entirely, but he has been more successful by well-regulated gymnastic movements of the wasted limb. For this purpose various ingenious instruments have been devised, which our space will not allow us to particularize.

